# Estradiol Activates PI3K/Akt/GSK3 Pathway Under Chronic Neurodegenerative Conditions Triggered by Perinatal Asphyxia

**DOI:** 10.3389/fphar.2018.00335

**Published:** 2018-04-09

**Authors:** G. Ezequiel Saraceno, Maria J. Bellini, Luis M. Garcia-Segura, Francisco Capani

**Affiliations:** ^1^Laboratorio de Citoarquitectura y Plasticidad Neuronal, Instituto de Investigaciones Cardiológicas “Prof. Dr. Alberto C. Taquini”, Consejo Nacional de Investigaciones Científicas y Técnicas, Universidad de Buenos Aires, Buenos Aires, Argentina; ^2^Interdisciplinary Institute for Neuroscience, Centre Broca Nouvelle-Aquitaine, UMR 5297, Université de Bordeaux, Bordeaux, France; ^3^Instituto de Investigaciones Bioquímicas de La Plata, Consejo Nacional de Investigaciones Científicas y Técnicas, Universidad Nacional de La Plata, La Plata, Argentina; ^4^Instituto Cajal, Consejo Nacional de Investigaciones Científicas y Técnicas, Madrid, Spain; ^5^Centro de Investigación Biomédica en Red de Fragilidad y Envejecimiento Saludable, Instituto de Salud Carlos III, Madrid, Spain; ^6^Universidad Autónoma de Chile, Santiago, Chile

**Keywords:** hippocampus, neuroprotection, western blot, signaling pathway, neuronal survival

## Abstract

Perinatal asphyxia (PA) remains as one of the most important causes of short-term mortality, psychiatric and neurological disorders in children, without an effective treatment. In previous studies we have observed that the expression of different neurodegenerative markers increases in CA1 hippocampal area of 4-months-old male rats born by cesarean section and exposed for 19 min to PA. We have also shown that a late treatment with 17β estradiol (daily dose of 250 μg/kg for 3 days) was able to revert the brain alterations observed in those animals. Based on these previous results, the main aim of the present study was to explore the mechanism by which the estrogenic treatment is involved in the reversion of the chronic neurodegenerative conditions induced by PA. We demonstrated that estradiol treatment of adult PA exposed animals induced an increase in estrogen receptor (ER) α and insulin-like growth factor receptor (IGF-1R) protein levels, an activation of the phosphatidylinositol 3-kinase/Akt/glycogen synthase kinase 3 beta/β-catenin signaling pathway and an increase in Bcl-2/Bax ratio in the hippocampus in comparison to PA exposed animals treated with vehicle. Taking together, our data suggest that the interaction between ERα and IGF-IR, with the subsequent downstream activation, underlies the beneficial effects of estradiol observed in late treatment of PA.

## Introduction

During perinatal lifetime, different factors selectively impair certain aspects of neurodevelopment, having long-lasting deleterious consequences in adult brain functioning (Basovich, 2010). Developing brains are susceptible to hypoxia-ischemia insults (obstetrical problem known as PA), which are associated with neurodevelopmental disorders (NDDs) (van Handel et al., 2007). The combination of an elevated vulnerability of the immature brain and the long-consequences in life quality, together with the limited availability of therapeutic tools to attenuate brain damage, supports the search for new preventive and therapeutic strategies (Manthey and Behl, 2006). During the last decade, the neuroprotective effects of the ovarian hormone 17β estradiol has been studied by several groups in different experimental pathological conditions (Bourque et al., 2009; Garcia-Segura and Balthazart, 2009; Kipp and Beyer, 2009; Kruse et al., 2009; Lebesgue et al., 2009; Pike et al., 2009; Suzuki et al., 2009). Previous studies from our laboratory showed that the ovarian hormone is able to reduce, in adult animals, chronic reactive astrogliosis and neuronal alterations in the hippocampus caused by PA (Saraceno et al., 2010). However, it is still unknown how estradiol triggers these reparative effects.

Most of the estradiol neuroprotective effects in the brain are mediated by activating several complementary signaling pathways by ERs (Arevalo et al., 2015). Classical ERs are transcription factors and two main isoforms, ERα and ERβ, have been characterized in mammals (Walter et al., 1985; Kuiper et al., 1996). Besides the ligand-binding domain, those ERs could be regulated by kinases activated by signaling pathways of several growth factors, such as IGF-1, due to their second activation domain (Ma et al., 1994; Mendez and Garcia-Segura, 2006). Finally, membrane-associated ERα and ERβ and non-classical ERs, such as G protein-coupled ER (GPER), also allow communication with the signaling cascade of other neuroprotective molecules (Ruiz-Palmero et al., 2013; Arevalo et al., 2015). Thus, considering the neuroprotective mechanisms of estradiol and ERs, it is important to recognize ERs as a key convergent point for estradiol signaling and the signaling of others neuroprotective factors.

Parallel neuroprotective mechanisms could be triggered in the brain by the activation of ERα, ERβ, and GPER. Moreover, ER activation also involves interactions with the protective pathways induced by other neuroprotective factors. On this regard, estradiol signaling and IGF-1 receptor (IGF-1R) signaling interact through ERα, allowing the formation of a multimolecular complex composed by ERα, IGF-1R and components of the IGF-1R signaling pathway such as, PI3K, Akt, and GSK3β (Mendez et al., 2003; Cardona-Gomez et al., 2004). This ERα/IGF-1R signaling interaction allows the regulation of PI3K-Akt-GSK3β-β-catenin signaling pathway by estradiol in the brain (Cardona-Gomez et al., 2004; D’Astous et al., 2006). It has been shown that inhibition of PI3K signaling abolishes the neuroprotective actions of estradiol in global cerebral ischemia and experimental stroke models (Jover-Mengual et al., 2010; Yang et al., 2010). Moreover, previous studies showed that estradiol and IGF-1 have a synergistic effect on Akt phosphorylation (Cardona-Gomez et al., 2002a), which regulates several transcription factors involved in the control of neuronal survival (Kane et al., 1999; Pugazhenthi et al., 2000). In addition, Akt activation by estradiol inhibits GSK3 activity promoting neuronal survival (Cardona-Gomez et al., 2004), inducing the activation of neuronal survival pathways (Cross et al., 1995) and decreasing tau protein phosphorylation (Cardona-Gomez et al., 2004).

Growing body of evidence postulates that the neuroprotective relevance of ERs depends on the implemented pathological model (Simpkins et al., 2012). Although we partially know how estradiol exerts neuroprotective actions in focal or global ischemia models when it was administrated before or shortly after the induction of the brain damage (Dubal et al., 2001; Carswell et al., 2004; Miller et al., 2005; Suzuki et al., 2007), the mechanism by which the hormone exerts reparative actions in the brain of adult animals exposed to PA (Saraceno et al., 2010) remain to be explored. Our main goals in the present study were to determine: (i) whether PA could affect the expression of ERs and their downstream signaling pathway activation, and (ii) whether estradiol treatment in adult animals exposed to PA exerts reparative actions by activating the neuroprotective mechanisms of ERs in parallel with the protective pathways induced by IGF-1.

## Materials and Methods

### Animals

All procedures involving animals were approved by the Institutional Animal Care and Use Committee at the University of Buenos Aires (CICUAL, #4091/04) and conducted according to the principles of the Guide for the Care and Use of Laboratory Animals (Animal Welfare Assurance, A-3033-01 protocol #S01084). Pregnant Sprague Dawley female rats and their male offspring were used in this study. Female rats in the 15th day of pregnancy were placed in individual cages and maintained on a 12:12 h light/dark cycle in a controlled temperature (21 ± 2°C) and humidity (65 ± 5%) environment. The animals had access to food (Purina chow) and tap water *ad libitum*. One group of animals (*n* = 10) was used as surrogate mothers, another group (*n* = 12) was assigned to PA procedures, and the remaining animals (*n* = 8) were the mothers of the control pups.

### Induction of Asphyxia

Twelve full-term pregnant rats on gestational day 22, were anesthetized (Saraceno et al., 2010), rapidly decapitated and the uterus horns were isolated through an abdominal incision and placed in a water bath at 37°C for 19 min (sub-severe PA) (Bjelke et al., 1991; Capani et al., 2001; Saraceno et al., 2010). Following PA, the uterine horns were rapidly opened, the pups were removed, the amniotic fluid was cleaned and the pups were stimulated to breathe by performing tactile intermittent stimulation with pieces of medical wipes for a few minutes until regular breathing was established. The umbilical cord was ligated and the animals were left to recover for 1 h under a heating lamp. When their physiological conditions improved, they were given to surrogate mothers that had delivered normally within the last 24 h. The different groups of pups were marked and mixed with the surrogate mothers’ normal litters (control animals that were left undisturbed). We maintained litters of 10 pups with each surrogate mother. Rats were weaned at 21 days of age and housed in groups of four animals per cage through the experiment. Only male animals were retained for the present study, while female animals were used for other research projects.

### Estradiol Treatment

**FIGURE 1:**
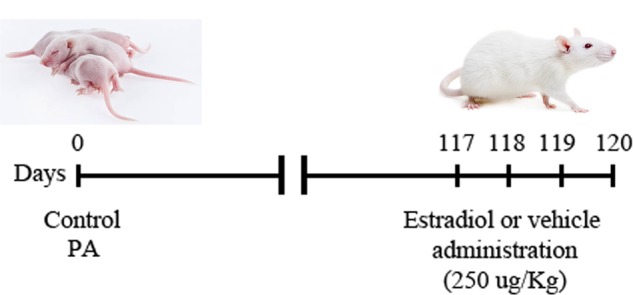
Schematic illustration of the experimental design. Dam rats that delivered no more than two pups (vaginally delivered controls, CTL) were hysterectomized, the uterus horns were immersed in a water bath at 37°C during 19 min [pups born by cesarean section plus asphyxia (PA)]. Intraperitoneal (i.p.) administration of 17β estradiol (250 μg/kg) or vehicle (0.9% saline solution) started 117 days after PA and it was repeated daily for 3 days consecutively (up to 119 days after PA). Animals were distributed in four experimental groups: (i) control animals injected with vehicle, (ii) control animals injected with 17β estradiol, (iii) animals submitted to PA and injected with vehicle and, (iv) animals submitted to PA and treated with 17β estradiol.

Thirty-two adult male rats were i.p. injected, 117 days after PA, with 17β estradiol (water soluble, E4389, Sigma, St. Louis, MO, United States; 250 μg/kg) or vehicle (0.9% saline solution) as described previously (Saraceno et al., 2010). The injections were repeated daily for 3 days consecutively (up to 119 days after PA) (**Figure 1**). Animals were distributed in four experimental groups: (i) control animals injected with vehicle, (ii) control animals injected with 17β estradiol, (iii) animals submitted to PA and injected with vehicle, and (iv) animals submitted to PA and treated with 17β estradiol.

### Immunoblotting

Sixteen male animals (120 days old; *n* = 4 per group) were killed by decapitation and the hippocampus was dissected. Subcellular fractionation was performed as previously described (Cardona-Gomez et al., 2002a,b). Briefly, dissected hippocampus was homogenized in a Dounce homogenizer with 1 ml of homogenization buffer (20 mM Tris-HCl, pH 7.4, 50 mM NaF, 1 mM Na_3_VO_4_, 1 mM EDTA, 1.25 μg/mL pepstatin A, 10 μg/mL leupeptin, 2.5 μg/mL aproptionin, 0.5 mM PMSF) containing 320 mM sucrose. Then, the homogenate was centrifuged at 1000 × *g* for 10 min and the supernatant was centrifuged at 16000 × *g* for 15 min to obtain the cytoplasmic cellular fraction. Protein concentration was estimated by Bradford technique (Bradford, 1976). The subcellular fractions were stored at -80°C.

Western blot analysis was carried out using cytosolic fractions separated on 8, 10, or 12.5% SDS-PAGE, in accordance with the molecular weight of the interested protein. Samples containing 20 μg of protein from each groups were applied to each lane. After electrophoresis (120 V for 90 min), proteins were transferred to polyvinylidene difluoride (PVDF) membrane for 2 h at 260 mA as described previously (Dunah et al., 1996). Cytoplasmic cellular fractions (*n* = 4 per group) were incubated overnight at 4°C with the following primary antibodies: anti-GPER (1:1000, mouse-IgG, Sigma-Aldrich), anti-ERα (mouse-IgG, 1:500, Sigma-Aldrich), anti-ERβ (mouse-IgG, 1:800, Sigma-Aldrich), anti-IGF-1R (rabbit-IgG, 1:1000, Santa Cruz), anti-Akt (1:1000, mouse-IgG, Cell Signaling), anti-GSK3-beta (1:500, mouse-IgG, Abcam), anti-GSK3 beta-phospho S9 (GSK3β-p-Ser, 1:500, rabbit-IgG, Abcam), anti-phospho-Akt-Thr308 (p-Akt, 1:1000, rabbit-IgG, Cell Signaling), anti PI3 (1:2000, mouse-IgG, Santa Cruz), anti β-catenin (1:1000, rabbit-IgG, Abcam). We used anti glyceraldehyde-3-phosphate dehydrogenase (GAPDH, 1:1000, rabbit-IgG, Sigma-Aldrich) as loading control. Blots were rinsed three times in PBS with 0.5% Tween-20 buffer (PBST), and then incubated with the corresponding horseradish peroxidase (HRP)-conjugated secondary antibody (1:1000, Bio-Rad) for 2 h at RT. Immunoreactive bands were detected using an ECL^TM^ Western Blotting Analysis System (Amersham^TM^, GE/Healthcare). Films were scanned and the optical density of protein bands was quantified using Gel Pro Analyzer software 3.1.00.00 (Media Cybernetics).

### Immunostaining, Confocal Microscopy, and Morphometric Analysis

Sixteen male animals (120 days old; *n* = 4 per group) were anesthetized with chloral hydrate (28% w/v; 0.1 ml/100 g body weight), and perfused intracardially with 4% paraformaldehyde in 0.1 M phosphate buffer, pH 7.4. Brains were removed and post-fixed in the same fixative solution for 2 h at room temperature and then immersed overnight at 4°C in 0.1 M phosphate buffer, pH 7.4. Coronal hippocampal sections (40 μm thick) were obtained using a Vibratome (VT 1000 S, Leica Microsystems, Wetzlar, Germany).

Immunofluorescence was performed on free floating sections under moderate shaking. Tissue sections were blocked for 30 min in phosphate-buffered saline (PBS) containing 0.3% bovine serum albumin (BSA; Sigma, St. Louis, MO, United States) and 0.3% Triton X-100. Sections were incubated overnight at 4°C with a rabbit polyclonal anti-GFAP antibody (1:2000, Sigma, St. Louis, MO, United States) and a mouse monoclonal anti-ERα antibody (mouse-IgG, 1:500, Sigma-Aldrich). After washing in buffer, tissue sections were incubated for 2 h at room temperature with Alexa 594 goat anti-rabbit IgG (1:200, Molecular Probes) and Alexa 488 goat anti-mouse IgG (1:200, Molecular Probes). Sections were counterstained with DAPI (Vector Laboratories, Inc., Burlingame, CA, United States) to label cell nuclei and mounted with Vectashield mounting medium. As specificity controls, some sections were incubated with the secondary antibody only. Under these conditions no immunostaining was observed. In order to minimize inter-assay variations, samples from all experimental groups were processed in parallel. Fluorescence was acquired on a commercial inverted confocal SPT-5 microscope (Leica Microsystems), with magnification of 63X oil DIC with excitation at 405, 488, 561 nm. Percentage of reactive area and colocalization analysis were estimated using Image J Program (Image J1.41o, NIH, United States). Briefly, images were obtained separately for each channel and analyzed. Single-channel images were thresholded and a binary image was created from each thresholded single-channel image. We measure the percentage of pixels in the selection that have been highlighted in red as an indicator of the percentage of reactive area. For colocalization analysis, binary images were then added together to display overlap, and a third binarized image of strict pixel overlap (colocalization) was used for analysis. Percentage of colocalization was calculated as colocalized ERα/GFAP divided by the total number of GFAP positive cells.

### Statistical Analysis

Material from eight rats was analyzed for each experimental group and for each parameter studied (total *n* = 32). All statistical analyses were performed by two-way analysis of variance (ANOVA) with birth condition (CTL and PA) and treatment (Vhi and 17β) as the main factors. When interaction effects were significant, analyses of the simple effects were carried out by *post hoc* comparisons using Student’s *t*-test (two-tailed) adjusted by Bonferroni correction. In any case neither the assumption of normal distribution (Shapiro–Wilk test) nor equality of variances (Levene’s test) was rejected. Results were expressed as the mean ± SEM. Differences with a *p* < 0.05 were considered to be significant. Statistical analyses were performed using the GraphPad Prism 5.03 statistical package for Windows (GraphPad software).

## Results

### Estradiol Increased the Expression of ERα in the Hippocampus of Adult Male Rats Subjected to PA

**FIGURE 2:**
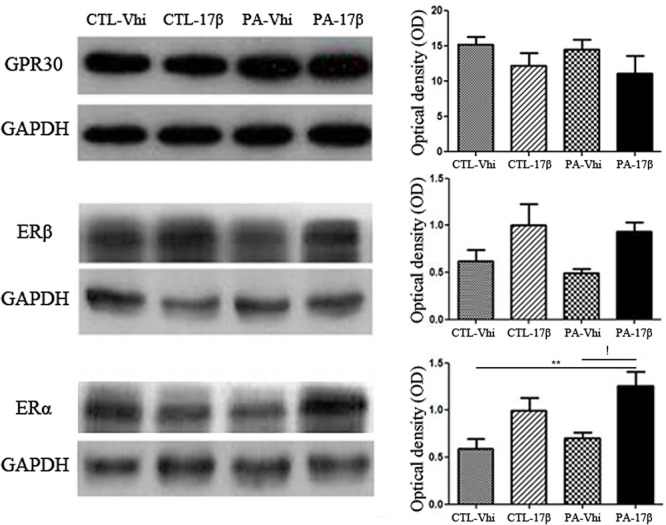
Hippocampal ERs expression analysis in adult male rats subjected to PA or treatment. Immunoblots were analyzed using different antibodies against GPER, ERβ, ERα, and GAPDH (loading control) in cytoplasmic fractions (*n* = 4 per group). The figure shows that PA does not induce any significant change regarding ERs and GPER level expression compared to control animals treated with vehicle or estradiol. In contrast, ERα protein level increased after the treatment with estradiol respect to asphyctic animals injected with vehicle and control animals treated with vehicle (!*p* < 0.05 PA-17β vs. PA-Vhi; ^∗∗^*p* < 0.01 PA-17β vs. CTL-Vhi after two-way ANOVA and Bonferroni *post hoc* test). Error bars represent the mean ± SEM.

As shown in **Figure 2**, the protein levels of ERα, ERβ, and GPER were not significantly different (*p* > 0.05) between the animals subjected to PA and injected with vehicle (PA-Vhi) and the control animals injected with vehicle (CTL-Vhi). Estradiol treatment induced a significant increase in the protein levels of ERα in PA exposed animals (PA-17β) (**Figure 2**), but did not affect the expression of ERβ and GPER (**Figure 2**).

**FIGURE 3:**
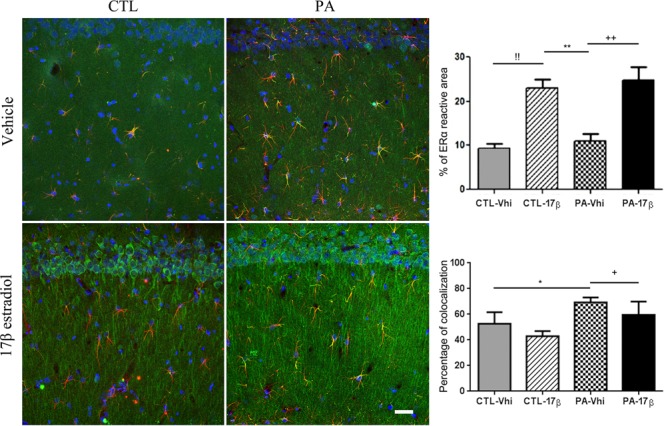
ERα immunoreactivity is modified by PA and estradiol treatment. Confocal microscope images of GFAP (red) and ERα (green) immunostaining from the *striatum radiatum* of CA1 hippocampal area (*n* = 4 per group). Percentage of ERα immunoreactive area was increased by estradiol in both control and estradiol treated animals. !! CTL-Vhi vs. CTL-17β; ^∗∗^*p* < 0.01, PA-Vhi vs. CTL-17β; ++*p* < 0.01, PA-Vhi vs. PA-17β after two-way ANOVA and Bonferroni *post hoc* test. Moreover, colocalization analysis revealed an increase in ERα immunoreactivity in astrocytes from PA exposed animals injected with vehicle respect to control group injected with vehicle and PA exposed animals treated with estradiol. Error bars represent the mean ± SEM. ^∗^*p* < 0.05, PA-Vhi vs. CTL-Vhi; +*p* < 0.05, PA-Vhi vs. PA-17β after two-way ANOVA and Bonferroni *post hoc* test. Scale bar: 10 μm.

Immunolocalization studies by confocal microscopy revealed ERα immunoreactivity in astrocytes in both vehicle and estradiol injected animals. The percentage of colocalization of ERα and GFAP immunoreactivities was increased in PA exposed animals injected with vehicle compared to control animals injected with vehicle. This suggests an induction in the expression of ERα in astrocytes by PA. The treatment with estradiol induced an increased ERα cytoplasmic immunoreactivity in CA1 pyramidal neurons and increases the percentage of ERα immunoreactive area in control and PA exposed animals (**Figure 3**).

### Estradiol Reverts the Effects of PA on IGF-1R/PI3-K/Akt/GSK3/β-Catenin Signaling Pathway

**FIGURE 4:**
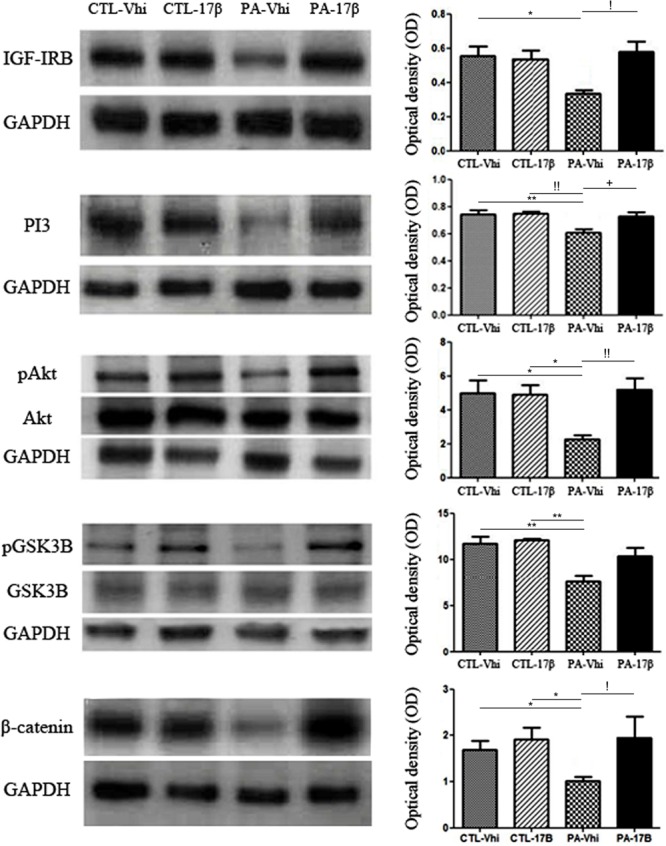
Perinatal asphyxia affects the IGF-1R/PI3-K/Akt/GSK3/β-catenin signaling pathway. Immunoblots were analyzed using different antibodies against IGF-IRB, PI3K, Akt, pAkt, GSK3B, pGSK3B, β-catenin, and GAPDH (loading control) in cytoplasmic fractions (*n* = 4 per group). The figure shows that PA affected the entire signaling pathway inducing a decrease in the protein expression/phosphorylation respect to control group injected with vehicle or estradiol (IGF-IRB: ^∗^*p* < 0.05 PA-Vhi vs. CTL-Vhi; PI3K: ^∗∗^*p* < 0.01 PA-Vhi vs. CTL-Vhi, !!*p* < 0.01 PA-Vhi vs. CTL-17β; pAkt/Akt: ^∗^*p* < 0.05; PA-Vhi vs. CTL-Vhi, PA-Vhi vs. CTL-17β; pGSK3B/GSK3B: ^∗∗^*p* < 0.01 PA-Vhi vs. CTL-Vhi, PA-Vhi vs. CTL-17β; β-catenin: ^∗^*p* < 0.05 PA-Vhi vs. CTL-Vhi, PA-Vhi vs. CTL-17β after two-way ANOVA and Bonferroni *post hoc* test). Estradiol reverted most of the changes induced by PA (IGF-IRB: !*p* < 0.05 PA-Vhi vs. PA-17β; PI3K: +*p* < 0.05 PA-Vhi vs. PA-17β; pAkt/Akt: !!*p* < 0.01 PA-Vhi vs. PA-17β; β-catenin: !*p* < 0.05 PA-Vhi vs. PA-17β after two-way ANOVA and Bonferroni *post hoc* test). Error bars represent the mean ± SEM.

Some of the estradiol effects on the brain are mediated by the interaction of ERs with the signaling of growth factors, such as IGF-1. We observed that PA decreased the expression of IGF-1 receptor, an effect that was reverted by estradiol treatment (**Figure 4**). Since estradiol treatment increased the levels of IGF-1R and ERα in PA exposed animals, we decided to study the effect of the hormone on the phosphatidyl inositol 3 kinase (PI3K) signaling pathway, which is downstream of IGF-1R. PA exposed animals showed a significant decrease in the protein levels of PI3K compared to the control groups (CTL-Vhi and CTL-17β). Estradiol treatment increased the expression of PI3K in PA exposed animals (PA-17β) to control levels (**Figure 4**).

The PI3K enzyme promotes the phosphorylation of the serine-threonine kinase enzyme Akt and its activation. In turn, Akt phosphorylates and inhibits GSK3β, which regulates the levels of β-catenin. No significant differences in total Akt and GSK3β protein levels were found between the experimental groups (**Figure 4**). However, PA induced a marked decrease in Akt phosphorylation (pAkt), determined as the ratio between its phosphorylation level at Ser473 and total Akt level (**Figure 4**). Estradiol treatment significantly reversed the downregulation of pAkt levels induced by PA (**Figure 4**). Moreover, PA decreased the levels of phosphorylated GSK3β (pGSK3β) and the levels of β-catenin and these effects of PA were reverted by estradiol (**Figure 4**). Together, all these findings suggest that PA induced a permanent downregulation of the PI3-K/Akt/GSK3/β-catenin survival pathway in the hippocampus and that estradiol treatment recovers this pathway.

### Estradiol Reverts the Effect of PA on Bcl-2/Bax Ratio

**FIGURE 5:**
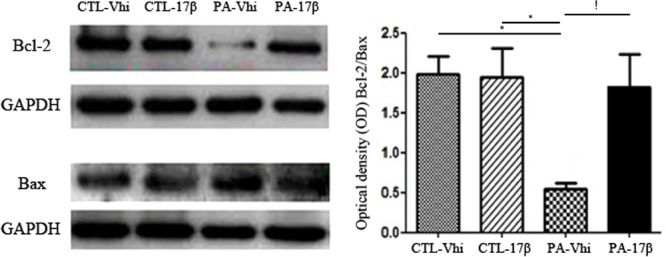
Bcl-2/Bax ratio is affected by PA and estradiol treatment. Immunoblots were analyzed using different antibodies against Bcl-2, Bax, and GAPDH (loading control) in cytoplasmic fractions (*n* = 4 per group). The figure shows that Bcl-2 expression was decreased under PA compared to control groups treated with vehicle or estradiol, affecting the Bcl-2/Bax ratio. Estradiol treatment increased the expression of Bcl-2, reverting the effects of PA (^∗^*p* < 0.05; PA-Vhi vs. CTL-Vhi, PA-Vhi vs. CTL-17β; !*p* < 0.05 PA-Vhi vs. PA-17β after two-way ANOVA and Bonferroni *post hoc* test). Error bars represent the mean ± SEM.

Given the changes in Akt caused by PA and estradiol treatments, we evaluated the levels of Bcl-2 and Bax. PA induced a decrease in Bcl-2 protein levels. In contrast, estradiol induced a significant increase in Bcl-2 protein expression. Bax protein levels were unaffected by the treatments (**Figure 5**). Together, all these findings indicate that PA decreased the Bcl-2/Bax ratio, while estradiol treatment significantly increased this ratio, suggesting a rescue response against the asphyctic insult.

## Discussion

Our findings indicate that adult rats submitted to PA present a decrease in the expression of IGF-1R, PI3K, β catenin, and Bcl2 and a decreased phosphorylation of Akt and GSK3β in the hippocampus. These changes observed in adult rats suggest that PA induces a permanent alteration in hippocampal IGF-1R signaling (Lee et al., 1996; Kartal et al., 2016). Interestingly, estradiol administration to adult rats submitted to PA was able to revert the decrease in the levels of IGF-1R, PI3K, pAkt, β-catenin, and Bcl2 in the hippocampus, suggesting that the IGF-1R/PI3K/Akt/GSK3β/β catenin signaling pathway is involved in the hormonal reversion of hippocampal neural deficits caused by early brain damage (Saraceno et al., 2010). These findings suggest potential new therapeutic targets for neuroprotection in infants who suffer PA.

Previous studies have shown that ERα is critical to determine the ability of physiological levels of estradiol to exert neuroprotection (Dubal et al., 2001). ERα mRNA levels are increased shortly after the induction of ischemic injury both in the presence and absence of estradiol (Dubal et al., 1999). In addition, estradiol induces the expression of IGF-1 (Kapur et al., 1992; Michels et al., 1993) and IGF-1R (Cardona-Gómez et al., 2000; El-Bakri et al., 2004) in the brain and activates the signaling cascade of IGF-1R via ERα (Kahlert et al., 2000; Marin et al., 2009). On the other hand, IGF-1R activation is essential for several actions of estradiol in the brain, such as neuroprotection in the hippocampus (Garcia-Segura et al., 2000). It has been shown that estradiol induces the formation of a multimolecular complex composed by ERα, IGF-1R and components of the IGF-1R signaling pathway in the brain (Mendez et al., 2003; Cardona-Gomez et al., 2004). This complex may be involved in the synergistic activation of Akt by IGF-1 and estradiol in the brain through ERα (Cardona-Gomez et al., 2002b).

Taken in consideration the previously discussed interaction of ERα with IGF-1R signaling in the brain, our present results may offer a mechanistic explanation for the reparative effects of estradiol in the hippocampus of adult animals exposed to PA. Thus, estradiol increased the expression of ERα in the hippocampus of adult animals that were submitted to PA. This may facilitate the activation of neuroprotective signaling pathways mediated by ERα (Arevalo et al., 2015). Indeed, estradiol restored the expression or phosphorylation of components of the PI3K/Akt/GSK3β/β-catenin signaling pathway that were decreased by PA. The restoration of this signaling pathway by estradiol may have direct consequences in the reparative process, including the reversion of dendritic and cytoskeletal alterations (Saraceno et al., 2010). Indeed, Akt activation regulates estradiol-induced synaptic plasticity (Znamensky et al., 2003) and is involved in the neuroprotective effects of the hormone (Zhang et al., 2001; Yu et al., 2004). Moreover, Akt activation modulates GSK3β activity (Cardona-Gomez et al., 2004), which regulates microtubule dynamics, neuritic growth, synaptogenesis, and synaptic plasticity (Hall et al., 2000). Therefore, estradiol regulation of GSK3β phosphorylation in the hippocampus of PA exposed animals may be involved in the reparative process. Furthermore, sustained activation of GSK3β may result in degenerative damage (Bhat et al., 2000), while its inhibition, regulated by estradiol (Cardona-Gomez et al., 2004), is associated with the activation of neuronal survival pathways in the hippocampus (Cross et al., 1995).

In this regard, inhibition of GSK3β activity by the *Wnt* signaling pathway has been shown to allow nuclear translocation and transcriptional activity of β-catenin, whereas GSK3β activation redirects β-catenin into the proteasome pathway (Barth et al., 1997). On the other hand, the activation of Akt by estradiol in the hippocampus of animals exposed to PA may also affect the expression of molecules that regulate apoptosis. Thus, we observed an increase in the relative concentration of Bcl-2 and a decrease in Bax, affecting the Bcl-2/Bax ratio in the hippocampus of PA exposed animals treated with estradiol. It has been shown that Akt activation also promotes phosphorylation of BAD, a member of the Bcl-2 family, and can suppress BAD-induced cell death (del Peso et al., 1997). Furthermore, the activation of Akt increases the activity of the Bcl-2 promoter, and estradiol induces the expression of Bcl-2 in neurons (Garcia-Segura et al., 1998; Pugazhenthi et al., 2000) by IGF-1R activation in the adult brain (Cross et al., 1995). Thus, the increase in Bcl2 expression in the hippocampus of PA exposed rats treated with estradiol may be a consequence of the restoration of Akt signaling and may also contribute to the reparative process.

Based on these encouraging findings, further experiments are needed to determine whether the effect of estradiol is permanent or transient since we just analyzed one single time point after the estradiol administration. In this line, since only male rats were included in our analysis, it will be important to examine the effects of PA and estradiol in females, because sex differences have been reported in the outcomes of different forms of perinatal brain injuries (Nuñez, 2012). Although these findings could help to determine a potential new therapeutic target for neuroprotection in infants, we know that the use of estradiol as therapeutic treatment is limited by its peripheral hormonal effects, such as increasing the risk of breast, endometrial, and ovarian cancer. Therefore, further studies should determine whether other estrogenic compounds that are free of these limitations, exert brain reparative actions after PA.

## Conclusion

**FIGURE 6:**
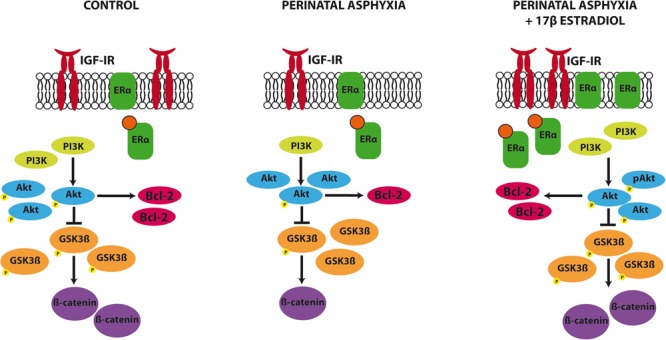
Summary of the changes induced by PA and estradiol treatment in the IGF-1R signaling pathway. PA induced a decrease in IGF-1R, PI3K, β catenin, and Bcl2 expression and a decrease in the phosphorylation of Akt and GSK3β in the hippocampus. Estradiol administration to adult rats submitted to PA was able to revert these changes, restoring the protein levels to similar values to those seen in control animals.

Our findings indicate that PA affects one key signaling pathway that promotes neuroprotection and modulates neuronal plasticity, the IGF-1R/PI3K/Akt/GSK3β/β catenin signaling pathway (**Figure 6**). The rescue of this signaling pathway by estradiol may contribute to the reparative actions of the hormone in the hippocampus of adult animals exposed to PA.

## Author Contributions

GS contributed to the conception and design of the research, performed the experiments, analyzed the data, and wrote the manuscript. MB performed data analysis and provided insightful ideas. LG-S contributed to the design, provided insightful ideas, wrote the manuscript, and provided financial support. FC provided financial support for animal housing.

## Conflict of Interest Statement

The authors declare that the research was conducted in the absence of any commercial or financial relationships that could be construed as a potential conflict of interest.

## Abbreviations

Akt: serine/threonine protein kinase
ER: estrogen receptor
GSK-3: glycogen synthase kinase 3
IGF-IR: insulin-like growth factor-I receptor
PA: perinatal asphyxia
PI3K: phosphoinositide 3-kinase
